# Innovations in Stress Urinary Incontinence: A Narrative Review

**DOI:** 10.3390/medicina61071272

**Published:** 2025-07-14

**Authors:** Tamas Szabo, Melinda-Ildiko Mitranovici, Liviu Moraru, Dan Costachescu, Laura Georgiana Caravia, Elena Bernad, Viviana Ivan, Adrian Apostol, Mihai Munteanu, Lucian Puscasiu

**Affiliations:** 1 Doctoral School, University of Medicine, Pharmacy, Sciences and Technology “George Emil Palade”, 540142 Targu Mures, Romania; szabo_tamas91@yahoo.com (T.S.); liviu.moraru@umfst.ro (L.M.); puscasiu@gmail.com (L.P.); 2 Department of Obstetrics and Gynecology, Emergency County Hospital Hunedoara, 14 Victoriei Street, 331057 Hunedoara, Romania; 3 Department of Radiology and Medical Imaging, “Victor Babes” University of Medicine and Pharmacy, 2 Eftimie Murgu Sq., 300041 Timisoara, Romania; costachescu.dan@umft.ro; 4 Faculty of Medicine, “Carol Davila” University of Medicine and Pharmacy, 050474 Bucharest, Romania; roman.laura.caravia@umfcd.ro; 5 Department of Obstetrics and Gynecology, Center for Laparoscopy, Laparoscopic Surgery, In Vitro Fertilization, “Victor Babes” University of Medicine and Pharmacy, 2 Eftimie Murgu Sq., 300041 Timisoara, Romania; bernad.elena@umft.ro; 6 Clinic of Obstetrics and Gynecology, “Pius Brinzeu” County Clinical Emergency Hospital, 300723 Timisoara, Romania; 7 Department of Cardiology “Victor Babes” University of Medicine and Pharmacy, 2 Eftimie Murgu Sq., 300041 Timisoara, Romania; ivan.viviana@umft.ro (V.I.); adrian.apostol@umft.ro (A.A.); 8 Faculty of Electrical Engineering, Technical University, George Baritiu Street, 400394 Cluj-Napoca, Romania; mihai.munteanu@ethm.utcluj.ro

**Keywords:** stress urinary incontinence, innovation in urogynecology, urinary incontinence in women, diagnosis, therapy

## Abstract

Urinary incontinence is characterized by the involuntary leakage of urine. The primary cause of stress urinary incontinence in women is the weakening of the pelvic floor muscles. Stress urinary incontinence (SUI) is a significant global health problem that impacts mainly middle-aged women, with a severe impact on their quality of life. Traditional diagnostic methods and treatments often fail, although technological innovations have improved diagnostic accuracy, such as specific questionnaires or transperineal ultrasound. While medical therapies and surgical procedures are continuously being developed, controversies about the correct choices regarding diagnostic and treatment methods continue to exist. The aim of our review was to identify the innovative diagnostic tools and effective treatment procedures for SUI. A narrative review was conducted due to the heterogeneity of the studies. New methods for diagnosis and treatment have gained ground, and we have covered them in our review; however, the field continues to expand. A personalized approach to diagnosis is also a requirement because of the limitations of conventional urodynamic studies, and we emphasize the importance of such personalization in enhancing clinical decision making. Future medical strategies that combine both preventive and therapeutic care are desirable. Newer technologies were brought to light in this review, including stem cell therapy and laser therapy.

## 1. Introduction

Stress urinary incontinence (SUI) is a significant global health problem that impacts mainly middle-aged women, with severe impact on their quality of life [1,2]. The reported prevalence ranges from 25% to 51% [3]. Urinary incontinence is characterized by the involuntary leakage of urine [4]. The primary cause of stress urinary incontinence in women is the weakening of the pelvic floor muscles. Intrinsic sphincter deficiency is another major cause, followed by urethral hypermobility and vaginal atrophy, common in menopausal patients [5].

Female pelvic floor dysfunction consists of clinical conditions, such as female pelvic organ prolapse and urinary and fecal incontinence, which can be associated with sexual dysfunction. The etiology and pathophysiology of these conditions are, however, not well understood [5,6,7]. The pathophysiology of stress urinary incontinence is multifactorial, with the contraction of the levator ani muscle and the external urethral sphincter being the most affected [5].

The classical epidemiology of SUI encompasses risk factors such as lifestyle and environmental factors, including age, parity, increased body mass index (BMI), the presence of diabetes mellitus, vaginal delivery, increased abdominal pressure, pelvic surgery, connective tissue disorders, and neurological conditions [5,8,9,10,11,12]. The principal causative factors remain childbirth and obstetric injury [13] as they induce alterations in the anatomical components responsible for continence [5].

Traditional diagnostic methods and treatments often fail [4]. The first American Urological Association guidelines proposed a dataset for the surgical management of female SUI in 1997, but recommendation adherence has been suboptimal [14]. Diagnostic and treatment modalities have also changed over the years [4].

Among the imaging techniques available, such as sonography, X-ray, computed tomography, and magnetic resonance imaging, ultrasound is superior for pelvic floor imaging, especially transperineal imaging. The technique is safe, cost-effective, easily accessible, and of high resolution [6,15]. Transperineal ultrasound is useful for determining residual urinary volume, bladder neck mobility, and other signs relevant in assessing pelvic organ prolapse and SUI, as well as for visualizing modern slings and mesh implants [6].

The International Urogynecological Association (IUGA) members’ recommendations for the management of urinary incontinence and pelvic organ prolapse are tension-free vaginal tape, Burch colposuspension, suburethral slings with low pressure, and bulking agent injection therapy [16]. However, traditional reconstructive methods are performed by the majority of surgeons [16] as treatment depends on their clinical management capabilities, so the recommended procedures are often underutilized. The same situation has been encountered in diagnostic procedures [15].

Due to its complex anatomy and function, the pelvic floor should not be divided into separate anterior, middle, and posterior compartments; it must be approached with a unitary vision. Urogynecologists, urologists, radiologists, and surgeons must form a multidisciplinary team to approach the treatment of the pelvic floor in an integrative way. Endovaginal, transperineal, and endoanal ultrasound approaches may be performed. Technological innovations, such as high-resolution 3D or 4D ultrasound, with new data post-processing characteristics and software options, can increase the accuracy of diagnosis. The imaging approach often differs among specialties, and standardization is needed [17,18]. Appropriate and timely investigations are mandatory to achieve better coordination of patient-centered care, but integrative services can be problematic, especially when the adoption of new practices is required [13]. Technological innovations have improved the diagnostic accuracy of imaging modalities [9]. Medical therapies and surgical procedures have also continuously developed, although controversies regarding the choice of diagnostic and treatment methods continue to exist [7].

The use of a vaginal mesh in pelvic floor disorders has increased rapidly over the past two decades, but is accompanied by a high rate of complications. The mesh is not observable through the use of MRI and CT scans; transperineal ultrasound is the only imaging modality that can present the mesh clearly, which has revolutionized the imaging and management of vaginal mesh kit complications [19]. Because of these complications, the use of mesh in vaginal prolapse surgery has become controversial [7].

On the one hand, new ideas and innovations generate controversies, and on the other hand, controversies may lead to further innovations [7].

Traditional diagnostic methods often fall short, especially for refractory urinary incontinence. This is due to their limited scope for continuous assessment. There are also many therapeutic options to enhance SUI symptoms, but there remains a lack of high-quality outcomes. The aim of this review is to identify the innovative diagnostic tools and effective treatment procedures for SUI, in order to update the current knowledge.

## 2. Materials and Methods

A narrative review was conducted due to the heterogeneity of the studies. Different outcomes were sought by the researchers. The PubMed, Google Scholar, and Cochrane databases were used to select relevant articles. A total of 2650 papers were obtained using the following specific keywords: stress urinary incontinence, women, diagnostic, treatment, and urogynecology in the last decade (2016–2025). The inclusion criteria were based on the evaluation of innovative diagnostic or treatment methods. Only manuscripts written in the English language were evaluated. Other studies written in a language other than English were excluded from our data analysis. Duplicates, poorly designed studies, and research with missing data were also excluded after the initial search. Two authors then independently and manually screened the relevant articles based on PRISMA 2020 guidelines. We identified 97 potentially relevant articles. A limitation of our study is the common objective outcome evaluated by the researchers, as the high heterogeneity of the methods limits general recommendations.

Our investigation was based on the following aspects:

- Traditional management in the diagnosis and treatment of SUI;
- Innovation in the diagnosis of SUI;
- Innovation in the treatment of SUI.

## 3. Traditional Methods in the Diagnosis and Treatment of Stress Urinary Incontinence

When diagnosing SUI, it is important to enquire about symptoms, as further investigations may be necessary. Conservative management should be the first step in SUI treatment, followed by surgical methods if required.

### 3.1. Diagnosis

Anamnesis is crucial in SUI diagnosis. Drinking habits, urination frequency, and medical history details must be obtained as a starting point, along with an accurate physical exam, including a neurological exam. Following this, a urine sample is collected to test for infection, and a urinary stress test is conducted during which the patient coughs to exert stress. An ultrasound test can show how much urine is left in the patient’s bladder after urination. Urodynamics is usually used to measure the pressure in a patient’s bladder during filling and emptying, and can also be used to check for stress incontinence and the strength of the pelvic floor muscles. Cystoscopy is used to identify any conditions in the bladder that may be causing the patient’s symptoms [5].

### 3.2. Treatment Options

The management of SUI involves a combination of conservative measures, such as lifestyle changes, and sometimes medical interventions. The first line of treatment is pelvic floor exercises alongside weight loss, both of which exhibit efficacy in symptom improvement [20,21]. As obesity can contribute to urinary incontinence, weight loss interventions, as a classical therapy, offer a unique clinical opportunity to improve SUI symptoms [11]. Losing weight can reduce pressure on the bladder and the pelvic floor muscles. Preventing constipation is another helpful measure. Urinating on a schedule can help the bladder hold more urine, and pelvic floor exercises (Kegels) help to strengthen the pelvic floor muscles, sometimes with the help of a physical therapist. Electrostimulation can also improve bladder function [5].

Medical interventions: Pharmacotherapy and neuromodulation are conservative efficient therapies [22], and topical estrogen may help restore tissue strength in the vagina. Anticholinergics, such as oxybutynin, block muscarinic receptors in the smooth muscle of the bladder, leading to the inhibition of detrusor contractions. Similar bladder-relaxing effects can be obtained using beta-3 adrenergic agonists. Duloxetine is associated with the inhibition of serotonin and norepinephrine at the pudendal nerve [5,23]. Some medications, such as duloxetine, may be used off-label. Additionally, pessaries can be inserted into the vagina to support the urethra and bladder [5].

For patients who do not respond to conservative measures, surgical interventions, such as sling procedures or urethropexy, may be considered. Minimally invasive procedures can also be used, such as injecting bulking agents into the urethra for extra support or using radiofrequencies to thicken the urethra [5,20].

The challenge lies in selecting the right approach, which depends on the severity of the SUI and the patient’s preferences. It is wise to start with conservative measures and progress to more invasive treatments if necessary.

## 4. Discussion Innovation in Stress Urinary Incontinence

A patient-centered standardization strategy is needed in order to identify correlations among the risk factors, diagnostic tools, treatment methods, and patient outcomes. A data-driven, personalized approach for clinical decision-making is required, centering on interdisciplinary management [24,25,26].

New methods for diagnosis and treatment have gained ground; however, the field continues to expand [27].

### 4.1. Innovation in Diagnosis

Among the traditional diagnostic procedures used in SUI medical history, physical examination, uroflowmetry, invasive urodynamics, the cough stress test, the pad test, and bladder diaries are widely used, but they all have limitations in their diagnostic capability [10,20,28,29]. A review of the literature conducted by Holroyd-Leduc et al. revealed that physical examination and bedside tests appear to be of modest value in diagnosing stress urinary incontinence, while a bladder stress test may be more helpful [30].

The Incontinence Impact Questionnaire assesses pelvic floor function in a reproducible and valid way [31,32,33,34]. As physical activity is known to be involved in the development of SUI, a newly developed pad test was evaluated during high-risk sports activities in female athletes, yielding good results [35]. Rubin et al. concluded, after their research (2024), that the presence of SUI affects the quality of life even more for high performance swimmers if it is not a factor of abandonment of the sport [36]. Pelvic Floor Distress Inventory Questionnaire (PFDI-20) and the Pelvic Organ Prolapse/Urinary Incontinence Sexual Function Questionnaire (PISQ-12) are validated instruments that evaluate the symptoms in patients with SUI, as well as the impact on the psychological state and quality of life of women with SUI [37].

New symptoms were included in the SUI diagnosis related to the quality of life of the patients. Psychometric evaluation was made in the Grigoriadis et al. research (2013) on 100 women with pelvic floor disorder, using the quality of life questionnaires. Symptoms and their impact on daily activities and emotional well-being were assessed using the Pelvic Floor Impact Questionnaire, (PFIQ-7) and the Pelvic Floor Distress Inventory Questionnaire (PFDI-20). It has been found that these questionnaires are comprehensible and valid tools to use with patients complaining of pelvic floor disorders [38]. These symptoms have been improved by various surgical treatments, according to the observations of Guan and Han (2024) [39].

Sexual function was also assessed through the Pelvic Organ Prolapse/Urinary Incontinence Sexual Function Questionnaire (PISQ-12), a validated instrument that evaluates symptoms in patients with UI and/or POP [37,40]. It has been proved that sexual activity is also affected by pelvic floor disorder, with significant impact on patient quality of life. A total of 221 women with SUI completed the PISQ-12 questionnaire in the Levy et al. study, demonstrating a correlation between parity and age and higher rates of pelvic organ prolapse complaints [37]. A notable improvement in emotional well-being and sexual function was observed in 924 women who had undergone anti-incontinence surgery in the research of Clark et al. (2020) [41].

More research is needed, with clinical trials conducted on large populations, to improve the test’s ease of use and accuracy in clinical practice [32,33,42].

Transperineal ultrasound has gained importance as a diagnostic tool for pelvic floor disorders [24]. Several studies have highlighted the utility of this approach in detecting SUI, identifying several sonographic parameters, such as urethral diameter [43]. Despite advancements in imaging, a comprehensive model remains elusive [44].

Transperineal ultrasound is also the only imaging technique that can be used to evaluate the presence of mesh and its complications. Correlations between ultrasonographic mesh characteristics, such as the distance between the mesh and the bladder neck, and pelvic organ prolapse (POP) recurrence were analyzed and the value of ultrasound in mesh evaluation was demonstrated [45]. A novel system, using an oversized bag introduced into the vagina and filled with water, was developed for the evaluation of the distension properties of the vagina. Three-dimensional transperineal ultrasound was used for the evaluation of the biomechanical and structural properties of the vagina [46]. As a future perspective, a conceptual catheter device was used in another study for real-time monitoring through transperineal ultrasound that promises to shift toward non-invasive diagnosis and management [42].

Other diagnostic techniques employed include shear-wave elastography, which incorporates sound touch elastography and sound touch quantification to assess urethral elasticity and bladder neck descent in women with SUI, thus providing a foundation for personalized management [44]. But this method involves expensive training and the cost-efficacy of the method was not evaluated.

Recent research has identified non-invasive biomarkers, including oxidative stress indicators and inflammatory markers, but researchers have encountered limitations inherent in single-biomarker approaches [47]. Altered extracellular matrix metabolism, modifications in estrogen receptors, oxidative stress, muscle cell differentiation, and contractility were found to be associated with SUI. However, due to heterogeneity in the studies, the precise contribution of the associated genes in relation to SUI pathophysiology remains unclear [48]. N-telopeptide cross-linked collagen (NTx) and inflammatory biomarkers, such as interleukin 6 (IL-6) and interleukin 10 (IL-10), were assessed in SUI, and the researchers found that patients with lower baseline NTx per mg of creatinine were less likely to experience surgical failure. Studies are needed to validate NTx as a possible independent biomarker for SUI surgery outcomes [49]. Biomarkers reflecting inflammation and neuroinflammation have also been measured, with these data suggesting that matrix remodeling and neuropeptide mediation may be involved in SUI pathophysiological mechanisms and response to treatment [50]. Their sensitivity and specificity was not evaluated, which is an important limitation in their use.

Vaginal microbiota has been identified as a possible pelvic floor disorder biomarker that can also work as a potential diagnostic tool. The findings highlight *Lactobacillus* as recurrent microbiota. Additionally, *Gardnerella*, *Streptococcus*, *Staphylococcus*, and *Proteus* are frequently observed, and are suggested to have an influential role in female urological and reproductive health [51]. Many studies employing 16S rRNA sequencing have identified the microbiota associated with various UI subtypes [1]. Reduced *Lactobacillus* levels and increased *Gardnerella* prevalence has been observed. Altered microbiome profiles with reduced Lactobacilli have been correlated with symptom severity, suggesting their protective role in maintaining urinary health [1]. Limitations of the studies include variability in the microbiome profiling methods, small sample sizes, and insufficient causal evidence, emphasizing the need for further research [1]. We found conflicting data that require further investigation. While some researchers have identified *E. coli* coexisting with SUI symptoms [1], others have considered *Gardnerella* and *Streptococcus* the predominant bacterial infection in SUI [51]. *Lactobacillus* was found to be decreased in most of the studies [1,45,52]. Integrating the microbiome into a comprehensive health assessment and treatment framework may become a necessity [1,2].

The genetic implications in SUI raise questions regarding its pathogenesis. Genome studies have been conducted to identify these genetic implications [53]. Eleven genes seems to be overexpressed, with the most frequent being the skin-derived antileukoproteinase (*SKALP*/*elafin*) and the collagen type XVII alpha 1 chain (*COL17A1*). These findings lay the foundations for the development of novel targeted therapies and diagnostic markers with applicability in clinical practice [54]. Macrophage receptors with a collagenous structure (*MARCO*) are biologically plausible biomarkers associated with stress incontinence, given their roles in smooth muscle contraction [55]. Circulating urinary miRNA may also be useful as a biomarker, aiding in the selection of more effective target therapies [56]. In another study, the effects of microRNA (miR)-34a on extracellular matrix (ECM) metabolism in fibroblasts of SUI were investigated. MicroRNA-34a overexpression promoted autophagy, inhibited ECM degradation, and ameliorated SUI by suppressing nicotinamide phosphoribisyltransferase (Nampt) [56,57,58,59]. MicroRNA-93 has also been found to regulate collagen I expression in fibroblasts via calpain-2 in stress urinary incontinence [60]. The use of these genetic diagnostic methods is expensive.

Proteomics-based research offers a new strategy to elucidate the etiology of stress urinary incontinence. Proteins secreted from the inner wall of the bladder and the urethra found in urine might explain the processes involved in the genesis of SUI [8]. Vascular cell adhesion molecule 1 (VCAM-1) was the only protein from urine samples collected in one study that reached statistical significance as a differentiating protein [60,61].

In a further study, the transcriptomic features of the key cell types associated with stress urinary incontinence were assessed, including epithelial cells, fibroblasts, smooth muscle cells, and T cells. Myofibroblasts were found to be reduced—a characteristic that could be used for the prevention or treatment of stress urinary incontinence. Fibroblasts and T cells participate in intercellular communication, which is altered in SUI; in addition, the extracellular matrix (ECM) metabolism and immune inflammatory responses exhibit a synergistic role in SUI. The ECM and the immune microenvironment are dysregulated in stress urinary incontinence, and potential biomarkers and therapeutic targets have been identified for future research [62].

The implication of extracellular vesicles, secreted by mesenchymal stem cells, in SUI has been explored. Their role in the extracellular matrix remodeling in fibroblasts seems to have an effect on stress urinary incontinence [62,63]. These vesicles pave the way for new treatment options through their inhibitory effect on vaginal fibroblasts in female stress urinary incontinence [63,64].

Artificial intelligence (AI) has revolutionized medicine and enhanced disease diagnosis accuracy, particularly in urogynecological disorders, by identifying patterns in vast datasets [65]. It paves the way for disease classification and personalized treatments [65]. AI algorithms that use 2D transperineal ultrasound (TPUS) images can simplify the clinical process of diagnosing SUI [66,67]. There are challenges and limitations in the implementation of AI technologies, such as data quality, interpretability, and integration into clinical practice. Nevertheless, it opens up the possibility of more accurate, efficient, and personalized care for patients [68].

A significant heterogeneity of the methods used to evaluate SUI was identified, as well as a paucity of data related to the results obtained in the assessment of pre-operative severity in SUI. New standardized methods for the management of this disease are needed [33,69].

The 2016 American Urogynecologic Society Prolapse Consensus Conference identified the critical areas of need for future research, and the requirement for the development of a multidisciplinary, standardized approach to the diagnosis and personalized treatment of pelvic floor disorders [70]. Clinical research in POP should align more closely with the needs and preferences of patients [69]. These diagnostic methods are exemplified in Table 1.

### 4.2. Innovation in Therapy

It has been demonstrated that changes in lifestyle, behavioral and dietary modifications, and pelvic floor muscle training (PFMT) are comprehensive conservative therapy options in SUI, supported by robust evidence [34].

Surgical therapy should only be considered after conservative therapies have failed. The mid-urethral sling is the gold standard second-line option for the surgical treatment of stress urinary incontinence, according to the 2016 guidelines of the European Society of Urology and the 2017 position statement of the European Urogynaecological Association [20,21]. However, concerns regarding mesh-related complications persist [20]. Urethral bulking agents are recommended for elderly patients [21], although a systematic review of the literature showed that bulking agents are less effective than surgical procedures in SUI [70].

Traditional surgeries such as colposuspension and autologous fascial slings are alternatives supported by robust evidence, but come with a different adverse event profile. The management of severe SUI remains a challenge, and while the therapeutic options are numerous, there is still a lack of high-quality data [20], and new therapeutic strategies are needed to achieve a better balance between efficacy and adverse events [21].

The efforts of multidisciplinary teams, including pelvic floor specialists, urogynecologists, radiologists, and physiotherapists, is essential [71]. Among the more innovative techniques in SUI management, transurethral radio frequency treatment seems to be an effective conservative treatment with few side effects reported [71,72,73,74].

The use of mesh in pelvic floor disorder surgery is a controversial issue, as adverse events have been described. The efficacy and safety of this surgical procedure remains to be demonstrated, despite its growing popularity [71]. New lightweight meshes have been used in pelvic organ prolapse (POP) surgery, which may reduce complications such as mesh exposure, chronic pain, and dyspareunia [45,75,76]. The efficacy of anti-infective knitted surgical meshes has also been proven [77]. In order to improve safety and quality of care, the Australasian Pelvic Floor Procedure Registry published a consumer-friendly public report covering the issues related to devices and/or prostheses [78].

In their study (2024) on 80 patients who underwent colposacropexy (CS), 40 patients through mini-laparoscopic surgery, and 40 patients using robotic surgery, Billone et al. observed that the mini-laparoscopic approach was preferable over the robotic one. Operative complication rates were similar for both groups, with novel standards prioritizing patient care [79].

A large scale review on a total of 15,855 patients showed that mid-urethral slings are more effective than the Burch colposuspension, according to the review by Capobianco et al. [80].

The artificial urinary sphincter is another innovative treatment but has not yet been tested with controlled randomized clinical trials, which is why this technique is not largely recommended as therapy for SUI [81]; further investigations are recommended.

For refractory SUI, other conservatory treatments have shown efficacy, such as sacral neuromodulation [82].

Researchers have demonstrated that laser therapy may be a safe and effective conservative treatment option for women who seek minimally invasive treatment for the management of SUI. This is a non-surgical therapy with no serious adverse effects. The limitations of studies on this approach include small sample sizes [2,83,84]. According to another study, SUI improved at short-term follow up after vaginal laser therapy, but the cure rates were low [77]. A reduction of effect was observed after 24–36 months, without being quantified, which also is a limitation of this procedure [85]. Another innovative approach was to bring together mathematics and medicine to support patient decision to choose between tension-free vaginal tape (TVT) surgery and vaginal Erbium laser (VEL) treatment, based on a correlation of demographic factors, patient outcomes, and patient preference [24,86].

The effects of low-intensity extracorporeal shockwave therapy (LiESWT) on the changes in urine leakage in stress urinary incontinence (SUI) was also evaluated [87]. LiESWT significantly improved the quality of life of patients and could serve as a potentially novel and non-invasive treatment for SUI, according to the pilot study by Gaspar and Brandi (2017) [84]. This procedure increased the thickness of the urethral wall, improved the striated and smooth muscle content, restored the integrity of the urothelium [88], and attenuated the SUI symptoms on physical activity [60].

Because surgical treatments are accompanied by procedure-related complications and high costs, new treatments have been developed based on the molecular mechanisms of stress urinary incontinence. Recent progress has been made in areas such as stem cell therapy, exosome differentiation, and gene regulation [89].

The preliminary results of stem cell therapy have shown great potential in SUI therapy, with different stem cells being used. In one study, the plasticity of directly injected stem cells was verified in terms of proliferating and differentiating into functional cells in vitro [89]. Stem cells are capable of self-renewal, differentiation, and releasing chemokines essential for tissue generation [2,90,91]. Re-cellularization by several types of autologous somatic/stem cells has gained interest in many diseases as future regenerative medicine. There is a risk of aberrant cell populations development leading to non-functional scar [92]. Another limitation is that most of the research is limited to the cell or animal research stage [89,93]. Our findings cannot be generalized due to the lack of clear evidence of their effectiveness.

A novel regenerative strategy promotes cell differentiation using exosomes as membrane-wrapped microcontainers. Exosomes are able to transport proteins, mRNA, or cytokines to target cells. Exosomes secreted by stem cells can prevent tissue damage and repair damaged tissues [89,93,94,95,96].

Regulating the expression of genes in SUI to restore damaged tissue is a feasible approach to therapy. CRISPR/Cas9 technology has been used to restore urination in SUI rats [97]. The chemokine stromal derived factor-1 (SDF-1) can induce tissue regeneration and improve continence function in a female SUI rat model [98].

Fibroblasts are affected by oxidative stress and apoptosis upon mechanical injury in a mouse model of SUI [99]. Nuclear factor erythroid 2-related factor 2 (Nrf2) is an inducer of antioxidant genes. It can be used to reduce oxidative damage and apoptosis, thus becoming a novel treatment strategy for mechanical-trauma-related SUI [100].

Regenerative materials have emerged as a promising strategy in SUI treatment. Novel biomaterials have been developed, such as urethral bulking agents and nano-gel composites for tissue regeneration, although assessing these therapies’ safety and effectiveness is essential [101]. Hydrogel microneedles (MNs) have been used in transdermal drug delivery, and their high biocompatibility could be applied in SUI treatment in combination with collagen type I. This minimally invasive technology is useful for the treatment and prevention of mild to moderate SUI, but it was used in animal models [102].

Meanwhile, treatments that could reverse the pathological changes in SUI are being sought, as future perspective. Researchers have continued the exploration for safe and reliable treatments, such as regenerative therapy. Some of these procedures, for example low-intensity pulsed ultrasound (LIPUS), pulsed electromagnetic field (PEMF), or low-intensity extracorporeal shockwave therapy (LiESWT), have been shown to restore the pathological changes in SUI [103]. LIPUS improves the pelvic floor through smooth muscle regeneration due to the modulation of the immune microenvironment [104]. LIPUS activates the pathways related to sympathetic nerve excitation, improving the SUI symptoms [105]. The efficacy and safety of a pulsed electromagnetic field (PEMF) treatment was proven for pelvic floor training in women with vaginal atrophy and SUI [106,107]. Moreover, the short-term benefits of magnetic stimulation were demonstrated in the stimulation of pelvic floor muscle contractility [2]. According to Biondo et al. (2022), flat magnetic stimulation improved the quality of life of patients with SUI without risk [108]. The PelviSense device has also been developed that may be effective in reducing the symptoms of SUI [109].

Another non-invasive treatment involves platelet-rich plasma injections, which have been shown to be effective and safe, at least in the short term. This procedure could be offered as an alternative treatment for SUI [110]. Adverse events following the injection of platelet-rich plasma have not been reported [111]; however, these encouraging findings need further investigation with randomized controlled trials [2,110]. Autologous platelet-rich plasma injected locally is also an experimental treatment that need external validation regarding its effectiveness. The accessibility for this new technique is challenging [112].

Promising results have been reported after medical treatment, including Litoxetine, a selective serotonin reuptake inhibitor, at phase III trials, associated with pelvic floor training [2]. These therapies are presented in Table 2. Our findings cannot be generalized due to the lack of clear evidence of their effectiveness.

Preventive treatment remains an important part of SUI management. Weight loss and pelvic floor muscle training (PFMT) remain the first-line treatments for SUI [113]. Lifestyle and diet, which have impacts on many diseases, have shown potential in preventing SUI [114]. As preventive treatment options, a combination of innovative pelvic floor muscle training (iPFMT) and duloxetine have shown its efficacy in the Hagovska et al. study (2020) on 158 women with pelvic floor disorders [115]. In their study, Weinstein et al. used the Intravaginal Motion-Based Digital Health System (PDHS) compared to pelvic floor muscle training with significant success in a randomized trial [116]. Functional magnetic stimulation also could be used to enhance the pelvic floor training [2,117].

Our review differs from other publications by updating current knowledge regarding diagnosis, prevention, and treatment in a critical way.

## 5. Conclusions

Despite existing diagnostic tools and treatment options for the management of SUI, patients continue to experience symptoms or side effects from the existing therapies. Therefore, there is a requirement for research to identify more effective and tolerable treatment options for patients. A personalized approach to diagnosis is also a requirement due to the limitations of conventional urodynamic studies. We emphasize its importance in enhancing clinical decisions. Future medical strategies that combine both preventive and therapeutic care are desirable. Newer technologies were brought to light in this review, including stem cell therapy and laser therapy. This manuscript provides future perspectives regarding the management of female stress urinary incontinence.

## Figures and Tables

**Table 1:** Innovations in SUI diagnosis.

Manuscript	Diagnostic Method	Efficacy	Limitations
[31,32,33,34,35]	Incontinence Impact Questionnaire	Reproducible and valid. Good efficacy.	Needs more accuracy in clinical practice.
[33,43,45,60]	Transperineal ultrasound	Identifies several sonographic parameters: sensitivity, 82.1%; specificity, 73% for urethral diameter [43]. Only imaging technique effective for mesh evaluation.	Comprehensive model remains elusive, training requirements.
[47,48,50]	Biomarkers: oxidative stress indicators, inflammatory markers, estrogen receptors, NTx, IL-6, IL-10	Encouraging results.	Researchers encountered limitations, especially in single-biomarker approaches; low cost-efficacy. Heterogeneity in studies.
[1,2,51,52]	Vaginal microbiota	Potential diagnostic tool.	Variability in microbiome profiling methods, conflicting data. Small sample sizes. Insufficient causal evidence.
[53,54,55,56,57,58,59]	Genetic implications	Basis for the development of novel targeted therapies.	Heterogeneity in studies, expensive.
[8,59]	Proteomics: VCAM-1 or other proteins secreted from the inner wall of the bladder and the urethra	Promising biomarkers.	More research needed, expensive.
[60,61]	Dysregulated immune microenvironment		Small sample size.
[62,63,64]	EVs	Regenerative treatment options.	More research needed, expensive.

NTx—N-telopeptide cross-linked collagen; IL-6, IL-10—interleukin 6 and 10; EVs—extracellular vesicles.

**Table 2:** Innovations in SUI therapy.

Studies	Treatment Options	Efficacy	Limitations
[71,72,73,74]	Transurethral radio frequency treatment	Safe.	Small sample size, need external validation.
[75,76,77,78]	New lightweight meshes	Safe.	Heterogeneity in the studies, different outcome were measured, different types of mesh were used.
[2,83,84,85]	Laser therapy	Safe, effective, conservative.	Cure rates were low in long-term follow-up.
[60,87,88]	Low-intensity extracorporeal shockwave therapy (LiESWT)	Efficient non-invasive treatment.	Training requirements.
[89,90,91,92,93]	Stem cell therapy	Capable of self-renewal, differentiation. Regenerative medicine with good preliminary results.	Limited data, animal research only, non-functional scar, limited accessibility.
[89,94,95,96]	Exosomes	Regenerative medicine, good preliminary results.	Research in progress, need external validation.
[89,97,98]	Regulating the expression of genes	Restores damaged tissue.	More study needed, low cost-effectiveness.
[89,99,100]	Oxidative stress	Promising treatment strategy for mechanical-trauma-related SUI.	Need external validation.
[101,102]	Urethral bulking agents or nano-gel composites	Conservative treatment with biomaterials.	Effective only in moderate SUI.
[103,104,105]	Low-intensity pulsed ultrasound (LIPUS)	Modulation of immune microenvironment and sympathetic nerve excitation.	Training requirements, validation needed.
[2,103,106,107,108,109]	Pulsed electromagnetic field (PEMF)	Pelvic floor training.	Training requirements, validation needed.
[2,110,111]	Platelet-rich plasma injections	Non-invasive treatment, effective and safe.	Only short-term follow-up, need external validation, limited accessibility.

LiESWT—low-intensity extracorporeal shockwave therapy; LIPUS—low-intensity pulsed ultrasound; PEMF—pulsed electromagnetic field.

## Data Availability

Data are available from the archives at the Department of Obstetrics and Gynecology, Emergency County Hospital Hunedoara, 14 Victoriei Street, 331057 Hunedoara, Romania.

## Abbreviations

The following abbreviations are used in this manuscript:

SUI	Stress urinary incontinence
BMI	Body mass index
IUGA	International Urogynecological Association
MRI	Magnetic resonance imaging
CT	Computed tomography
POP	Pelvic organ prolapse
NTx	N-telopeptide cross-linked collagen
IL	Interleukin
ECM	Extracellular matrix
Nampt	Nicotinamide phosphoribisyltransferase
VCAM	Vascular cell adhesion molecule
TPUS	Transperineal ultrasound
AI	Artificial intelligence
PFMT	Pelvic floor muscle training
LiESWT	Low-intensity extracorporeal shockwave therapy
Nrf2	Nuclear factor erythroid 2-related factor 2
MNs	Hydrogel microneedles
LIPUS	Low-intensity pulsed ultrasound
PEMF	Pulsed electromagnetic field
